# Cerebellar Transcranial Direct Current Stimulation (ctDCS): A Novel Approach to Understanding Cerebellar Function in Health and Disease

**DOI:** 10.1177/1073858414559409

**Published:** 2016-02

**Authors:** Giuliana Grimaldi, Georgios P. Argyropoulos, Amy Bastian, Mar Cortes, Nicholas J. Davis, Dylan J. Edwards, Roberta Ferrucci, Felipe Fregni, Joseph M. Galea, Masahi Hamada, Mario Manto, R. Chris Miall, Leon Morales-Quezada, Paul A. Pope, Alberto Priori, John Rothwell, S. Paul Tomlinson, Pablo Celnik

**Affiliations:** 1 Unité d’Etude du Mouvement, ULB-Erasme, Brussels, Belgium; 2 Department of Psychology, Brain, Action and Cognition Lab, University of London, Egham, Surrey, UK; 3 Department of Neuroscience, Johns Hopkins University School of Medicine, Baltimore, MD, USA; 4 Burke Medical Research Institute, Departments of Neurology and Neuroscience, Weill Medical College of Cornell University, White Plains, NY, USA; 5 Department of Psychology, Swansea University, Swansea, UK; 6 Department of Medical-Surgical Pathophysiology and Transplants, University of Milan, Milan, Italy; 7 Clinical Center for Neurotechnology, Neurostimulation and Movement Disorders, Fondazione IRCCS “Ca’ Granda” Ospedale Maggiore di Milano, Milan, Italy; 8 Center of Neuromodulation, Spaulding Rehabilitation Hospital and Massachusetts General Hospital, Harvard University, Boston, MA, USA; 9 School of Psychology, University of Birmingham, Edgbaston, Birmingham, UK; 10 Department of Neurology, The University of Tokyo, Tokyo, Japan; 11 Institute of Neurology, University College London, London, UK; 12 School of Psychology, Bangor University, Bangor, UK; 13 Department of Physical Medicine and Rehabilitation, Johns Hopkins University School of Medicine, Baltimore, MD, USA

**Keywords:** transcranial, direct current stimulation, ctDCS, cerebellum, modeling, motor, plasticity, cognitive, working memory, emotion, language, safety, learning

## Abstract

The cerebellum is critical for both motor and cognitive control. Dysfunction of the cerebellum is a component of multiple neurological disorders. In recent years, interventions have been developed that aim to excite or inhibit the activity and function of the human cerebellum. Transcranial direct current stimulation of the cerebellum (ctDCS) promises to be a powerful tool for the modulation of cerebellar excitability. This technique has gained popularity in recent years as it can be used to investigate human cerebellar function, is easily delivered, is well tolerated, and has not shown serious adverse effects. Importantly, the ability of ctDCS to modify behavior makes it an interesting approach with a potential therapeutic role for neurological patients. Through both electrical and non-electrical effects (vascular, metabolic) ctDCS is thought to modify the activity of the cerebellum and alter the output from cerebellar nuclei. Physiological studies have shown a polarity-specific effect on the modulation of cerebellar–motor cortex connectivity, likely via cerebellar–thalamocortical pathways. Modeling studies that have assessed commonly used electrode montages have shown that the ctDCS-generated electric field reaches the human cerebellum with little diffusion to neighboring structures. The posterior and inferior parts of the cerebellum (i.e., lobules VI-VIII) seem particularly susceptible to modulation by ctDCS. Numerous studies have shown to date that ctDCS can modulate motor learning, and affect cognitive and emotional processes. Importantly, this intervention has a good safety profile; similar to when applied over cerebral areas. Thus, investigations have begun exploring ctDCS as a viable intervention for patients with neurological conditions.

## Introduction

Despite the importance of the cerebellum in both motor and cognitive behaviors, very few interventions have been developed that aim to modulate cerebellar function. In the past 5 years, there have been an increasing number of studies applying cerebellar transcranial direct current stimulation (ctDCS) to study the functions of the cerebellum. This non-invasive technique has generated significant interest in the neuroscience, neurology, and rehabilitation communities. ctDCS results in polarity-dependent, site-specific modulation of cerebellar activity and has therefore been used to understand the physiological role of the cerebellum during diverse cognitive and motor behaviors in alert humans. In addition, results so far suggest that ctDCS may represent a useful therapeutic intervention for patients with neurological conditions. Thus, ctDCS is bringing new hope to the field of cerebellar disorders, a constellation of conditions that currently lack clear and beneficial symptomatic and disease-modifying treatments. In this review, we discuss the mechanisms of action of ctDCS and highlight the most relevant published studies. We also discuss the safety issues related to the modulation of the cerebellar function.

## Can Direct Current Stimulation Affect Cerebellar Excitability?

**Figure 1.**
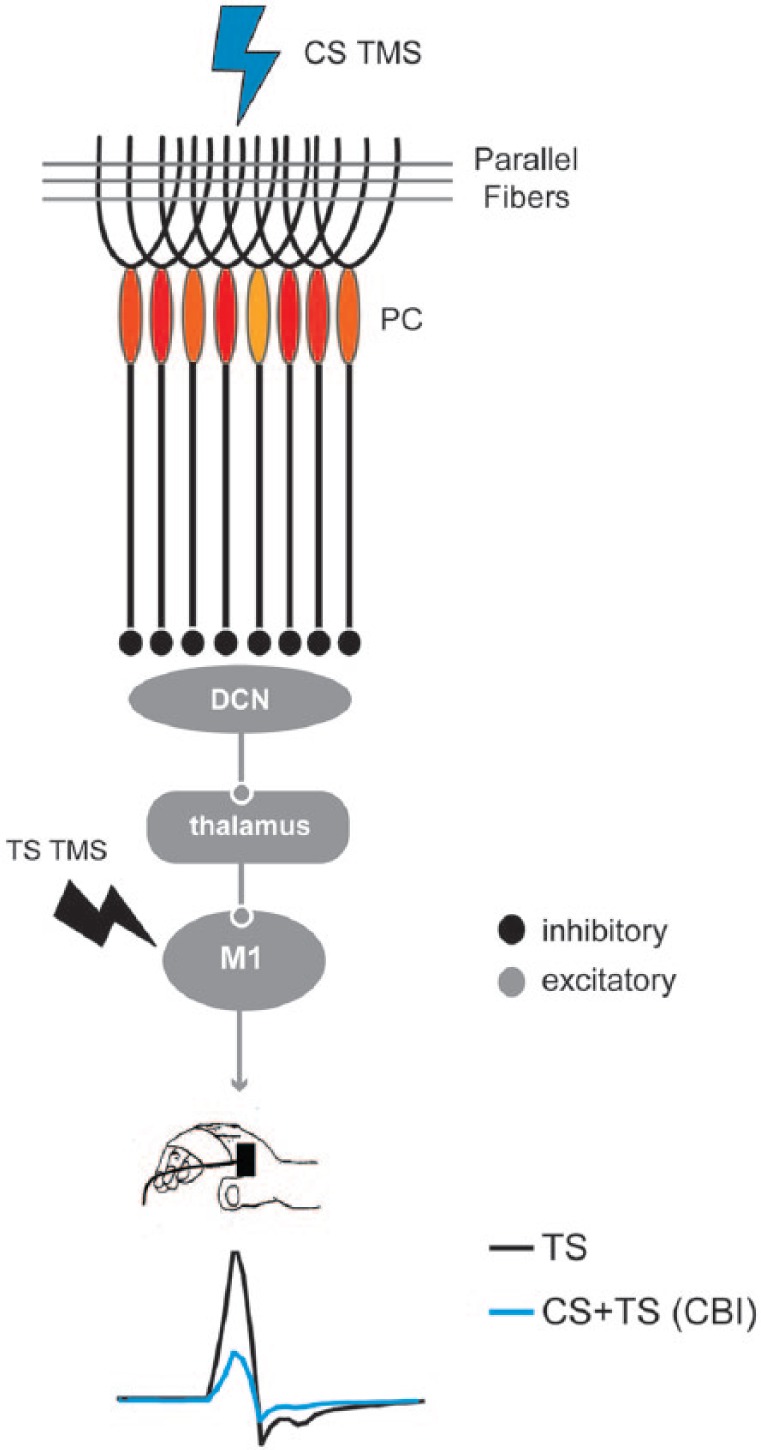
Cerebellar–primary motor cortex (M1) connectivity measure assessed by transcranial magnetic stimulation (TMS). The scheme represents the current interpretation of how the circuitry from the cerebellar cortex to M1 is engaged when assessed with TMS. A conditioning TMS pulse (CS, blue lightning) delivered over the cerebellum activates many Purkinje cells (PCs) leading to inhibition of the deep cerebellar nuclei (DCN). Since the DCN has a disynaptic excitatory connection to M1, inhibition of the DCN by PC stimulation leads to reduce excitation of M1. This inhibition is evidenced by the reduced amplitude of motor evoked potentials (MEPs) in response to test stimuli (TS, black lightning) to M1 when compared with unconditioned TS. The difference in MEP amplitudes (black–blue traces) represent the magnitude of inhibition the cerebellum (CB) is exerting over M1, a measured known as cerebellar inhibition or CBI. Please note that MEPs are recorded from a muscle typically from the hand (i.e., first dorsal interosseuos as shown in the figure) or leg using surface electromyography electrodes.

Objective and scientifically rigorous measures are required to ascertain whether the application of tDCS to the back of the head modulates cerebellar excitability. Similar to previous studies applying tDCS over cerebral areas (Nitsche and Paulus 2000, 2001), transcranial magnetic stimulation (TMS) was used to demonstrate that tDCS modulates cerebellar function. In the 1990s, Ugawa and other investigators showed that stimulating the cerebellum with directly-applied electrical current (Ugawa and others 1991) or TMS (Ugawa and others 1995; Weise and others 2006; Werhahn and others 1996) induced changes in amplitudes of motor evoked potentials (MEPs) elicited from the primary motor cortex (M1). These investigations demonstrated that the application of a conditioning TMS pulse over the cerebellum 5 to 7 ms prior to a TMS stimulus over M1 reduced the MEP amplitudes relative to single TMS stimuli over M1. This was interpreted to be due to activation of Purkinje cells (the sole output of the cerebellar cortex) by the conditioning pulse leading to inhibition of the dentate nucleus, which in turn has excitatory connections with M1 via the thalamus (Daskalakis and others 2004; Pinto and Chen 2001) (Figure 1). Because of the inhibitory nature the conditioning stimuli over the cerebellum has on M1 this effect was called cerebellar inhibition (CBI). CBI can be used as a neurophysiological technique to measure connectivity between the cerebellum and motor cortex (CB–M1 connectivity) and lends itself to the assessment of whether tDCS can modulate cerebellar excitability.

In 2009, Galea and colleagues demonstrated that it is possible to increase or decrease the connectivity between the cerebellum and M1 (Galea and others 2009), depending on the polarity of applied tDCS. The investigators delivered tDCS using an electrode over the same location targeted by TMS when measuring CBI and a reference electrode positioned ipsilaterally over the face. They found that ctDCS resulted in a polarity-specific modulation of CBI when the intensity was set at 2 mA for 25 minutes. Cathodal stimulation decreased the ability of TMS to elicit CBI of M1, whereas anodal tDCS, could do the opposite as long as low intensities of conditioning stimuli were used to elicit CBI and avoid “ceiling” effects. Importantly, several control experiments demonstrated that the modulation of CBI by tDCS was not accompanied by changes in spinal, brainstem, or motor cortex excitability. Thus tDCS over cerebellum could modulate the excitability of neurones involved in CBI. Noteworthy, the polarity-specific effects of tDCS over the cerebellum were later confirmed by behavioral studies, an example being the demonstration of the facilitation of locomotor adaptation by anodal tDCS and the converse after cathodal tDCS as shown by Jayaram and others (2012). These results taken with other studies (Galea and others 2011) that have controlled for modulation of adjoining areas, such as vestibular or visual cortex, and with investigations using finite element modeling (Rampersad and others 2013), suggest that the effects of ctDCS are caused by an action on the cerebellum rather than the cerebral cortex.

Altogether, the evidence indicates that tDCS can be applied to modulate the excitability of some elements in the cerebellum. This has presented the opportunity to investigate the role of the human cerebellum within different behaviors, and more relevant to patients, to develop this technique as a therapeutic tool.

## Does Cerebellar tDCS Affect Motor Cortex Plasticity?

It has been proposed that ctDCS is most likely to produce its effects by polarizing Purkinje cells (Galea and others 2009) and changing the levels/pattern of activity in the deep cerebellar output nuclei (see also next section). Given the many targets of cerebellar output, the question arises whether or not ctDCS might have an effect on distant structures such as the cerebral cortex. Given the dense anatomical connections (Middleton and Strick 2000), a number of authors have probed the influence of ctDCS on M1. Hamada and others (2012) argued that if the cerebellum plays an important part in motor learning then it may also affect the learning “rules” in other parts of the motor system such as M1. They therefore performed a study using paired associative stimulation (PAS), a protocol frequently employed to study M1 plasticity. PAS involves repeated pairing of an electrical stimulus to the median nerve with a later TMS pulse over the contralateral motor cortex (Stefan and others 2000). Application of around 100 pairs alters corticospinal excitability for about an hour after stimulation. In the PAS protocol, it has been shown that the interval between median nerve and TMS stimuli is critical: intervals of 21.5 to 25 ms increase motor cortex excitability whereas intervals of around 10 ms reduce excitability (Stefan and others 2000; Weise and others 2006; Wolters and others 2003, 2005). These effects together with pharmacological manipulations showing that PAS aftereffects are dependent on *N*-methyl-d-aspartate (NMDA) receptor activity (Stagg and others 2009; Stefan and others 2002; Wolters and others 2003), suggest that PAS elicits plasticity changes in M1 resembling long-term potentiation (LTP) and long-term depression (LTD) similar to spike timing dependent plasticity shown in animal models (Müller-Dahlhaus and others 2010).

The PAS paradigm, with its time-dependent effects, implicitly assumes that all sets of rapidly conducted dorsal column–medial leminiscal inputs are responsible for the plasticity induced by PAS at all timings. However, recent findings using ctDCS suggest that this may not be the case. Hamada and others (2012) found that ctDCS blocked induction of PAS25 aftereffects but not the effects of PAS at 21.5 ms (PAS21.5), both of which have been shown to induce a lasting increase of MEPs (i.e., LTP-like plasticity). Hamada and others argued that there are separate mechanisms mediating the effects of PAS at these two interstimulus intervals, where only the PAS25 effect depends on the cerebellum. This is because they failed to find changes on somatosensory evoked potentials and PAS21.5, thus excluding a potential thalamic or sensory cortex substrate of ctDCS. Of note, although ctDCS seems to modulate cerebellar excitability in a polarity specific manner (Galea and others 2009), both anodal and cathodal ctDCS blocked the PAS25 effects. Therefore, it is still possible that this effect was mediated by mechanisms other than cerebellar excitability changes. However, we think this is unlikely given that similar findings pointing to the ability of non-invasive brain stimulation techniques to modulate the cerebellar role on sensory processing were also observed when using cerebellar TMS (Popa and others 2013).

Taking these results together, ctDCS seems an effective method to modulate cerebellar excitability, which in turn can affect distant plasticity in human cortical areas (i.e., the motor cortex).

## What Are the Mechanisms of Action of ctDCS?

There are two important elements to be considered in forming an explanation for the mechanisms of action of ctDCS: whether the effects of ctDCS are polarity specific and whether ctDCS is delivered during a behavioral intervention (on-line effects) or before behavioral performance (off-line effects).

### Are the Effects of Cerebellar tDCS Polarity Specific?

Several studies that have used ctDCS to examine human cerebellar function have reported a polarity-specific effects (Galea and others 2009; Jayaram and others 2012) whereas other investigations found no differences between anodal and cathodal ctDCS (Shah and others 2013). The inconsistency of these results remains puzzling, but details on how the different studies were performed may shade some light to the discrepant findings. One of the important factors to be considered is the different behaviors that have been used to assess the effects of ctDCS. Research has focused on the effects of ctDCS on motor, cognitive and emotional behaviors, which likely rely on different cerebellar neural substrates. Since the orientation of neurons in different cerebellar areas varies in relation to the applied electric field, this may account for the seemingly differing effects of ctDCS on the different study domains. Furthermore, this issue is exacerbated if one considers the wide range of stimulation montages, electrodes size and parameters that have been employed so far. While several studies have used a large stimulating electrode covering the whole cerebellum (Ferrucci and others 2008, 2012, 2013), others used smaller electrodes designed to stimulate only one hemisphere (Block and Celnik 2013; Galea and others 2009; Jayaram and others 2012). The first set of studies (mostly in the cognitive and emotional domain) used a montage from the back of the head bilaterally to one shoulder found not polarity specific effects. The second group of studies (focused mostly on motor function) used a montage from one side of the back of the head (aiming at only one cerebellar hemisphere) to the ipsilateral face (over the buccinators muscle) found polarity specific changes.

Interestingly, recent investigations applying tDCS over the cerebral cortex have also described lack of polarity specific effects when the intensities are increased (Batsikadze and others 2013). Specifically, this study found that cathodal tDCS, which typically has been described to elicit reduction of M1 excitability when applied at 1 mA, resulted in increased excitability when delivered at 2 mA. While intensity seems to be another factor that can influence the effects of tDCS, most studies applying cerebellar tDCS, however, have used 2mA, including those that show polarity specific and non-specific effects. This is because the distance from the scalp to the cerebellum is larger than the one to reach M1. Therefore, although not completely understood, intensity cannot explain the discrepant findings across studies using ctDCS.

**Figure 2.**
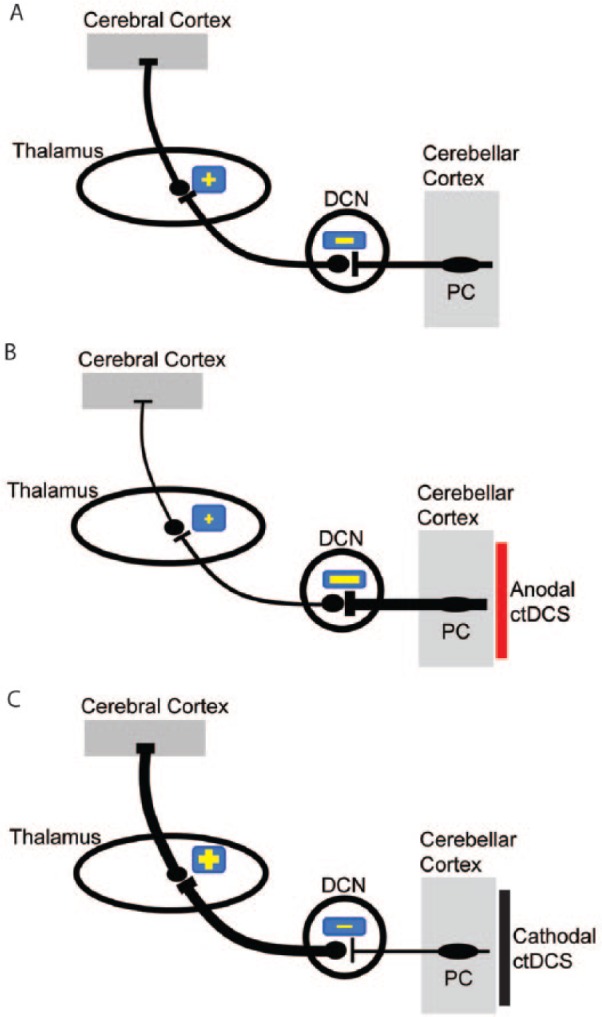
Illustration showing the current interpretation of how cerebellar transcranial direct current stimulation (ctDCS) affects the cerebellar–thalamocortical pathway. (A) Purkinje cell (PC; major neurons of the cerebellar cortex) exert physiologically an inhibition (−) over the deep cerebellar nuclei (DCN). These latter project on contralateral thalamic nuclei (+: excitatory thalamic pathway), which project themselves diffusely on the cerebral cortex. (B) Anodal ctDCS (red) is thought to increase the excitability of PC. In this model, the inhibition from the cerebellar cortex to DCN is augmented, hence reducing nucleothalamic facilitatory drive to cortical areas. (C) Cathodal ctDCS (black) decreases the activity of the cerebellar cortex. Thus, the DCN is disinhibited releasing the inhibition of the nucleothalamic drive.

Importantly, within TMS studies ctDCS effects have been found to be polarity specific in a study that applied the more focal hemispheric montage (Galea and others 2009). The consequences of ctDCS on CB–M1 connectivity were interpreted as resulting from a modulation of the activity of Purkinje cells and other neurons of the cerebellar cortex, ultimately affecting inhibition of the cerebellar nuclei (Figs. 1 and 2). While anodal ctDCS increased CBI, cathodal stimulation elicited the opposite effect (Galea and others 2009). This finding is thought to be due to modulation of the output of Purkinje cells with the consequent inhibition of the facilitatory pathway from cerebellar nuclei to cortex.

To date, there have been very few animal studies that have employed ctDCS. Early investigations conducted by Moruzzi and others in the 1950s and 1960s showed that the cerebellum is highly susceptible to polarizing currents (Gauthier and others 1955; Mollica and others 1953a, 1953b, 1953c; Pompeiano and Cotti 1959a, 1959b). While it is true that the cerebellum is subject to the same principles that govern the influence of electric fields on the rest of the central nervous system, it is possible that its intrinsic passive electrical properties (Nightingale and others 1983; Yedlin and others 1974) may modify its response to the application of electricity. Therefore, animal work remains scant in this area and of little help so far to elucidate the mechanisms underlying ctDCS.

### On-line versus Off-line ctDCS Effects

Another important consideration when discussing the mechanisms of ctDCS are the complex interactions likely to occur between the behavior being assessed and the effects of simulation. These need to be addressed separately in those studies delivering ctDCS as an “off-line” approach, in which behavior is tested after the end of several minutes of simulation (“aftereffects”), and in those that describe behavioral changes during stimulation (“on-line” effects).

### On-line Effects

Animal studies in the turtle show that electric fields affect many types of cerebellar neurons depending on their orientation with respect to the applied field (Chan and Nicholson 1986). Current flowing from the cortical surface to the fourth ventricle predominantly excites the cell bodies and proximal dendrites of Purkinje cells and hyperpolarizes the apical dendrites (Chan and Nicholson 1986). In these experiments, application of high field strengths of 15 to 20 mV/mm could even induce action potentials in the neurons. Much smaller fields of around 1 mV/mm are thought to be produced by ctDCS in humans (Parazzini and others 2013, 2014). However, the applied electric field could also affect other neurons, fibers, and glial cells.

**Figure 3.**
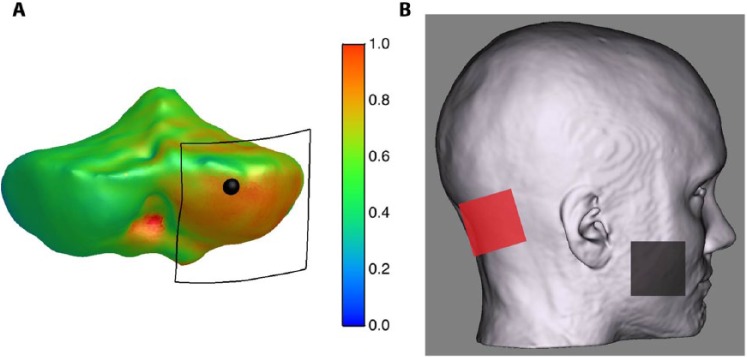
The figure shows the electrical field strength and distribution over the cerebellum (A) when a unilateral hemispheric montage is used (B). Note the position of the active electrode (black square) targeting the lateral cerebellum (black dot represents the actual target). When using this montage, cerebellar transcranial direct current stimulation (ctDCS) delivers a fairly unilateral high electrical strength over the anterior and posterior cerebellar hemisphere. Modified from Rampersad and others (2014).

To understand potential mechanisms of ctDCS in humans recent studies have focused on modelling the electrical field and the human head. The posterior and inferior aspects of the cerebellum are closest to the skull and therefore most accessible to transcranial stimulation. These areas are principally lobules VI to VIII and the crura of the posterior lobe. The surface curvature of the cerebellum restricts access to lobules IX and X, and the inferior sections of the vermis lie deep between the hemispheres. The anterior lobe and lobules I to V are likely too far from the scalp surface, although the high conductivity of cerebrospinal fluid and the choice of electrode placement mean that in principle current could be directed toward deeper targets (Miranda and others 2006). Preliminary attempts at modeling the electric field in ctDCS with a large electrode over cerebellum and a reference electrode on the ipsilateral shoulder suggest that the posterior aspect of the cerebellum and the posterior occipital lobe can be affected when delivering ctDCS (Ferrucci and others 2012; Parazzini and others 2014). However, more recent modelling investigations addressing the unilateral hemisphere montage used in several motor (Galea and others 2011; Jayaram and others 2012) and physiological (Galea and others 2009) studies, namely the active electrode over the lateral cerebellum (centered 3 cm from the inion) and a reference electrode over the ipsilateral buccinator muscle, concluded that the electrical fields are maximal in the targeted cerebellar hemisphere with little activation of other neural structures (Rampersad and others 2014) (Figure 3). Nonetheless, current human modelling work does not describe in detail the ctDCS effects on individual neural elements, the site (cerebellar cortex, cerebellar nuclei, or cerebellar white matter), the nature of changes or how they influence cerebellar functions. In addition, it is not clear whether its effects are mediated by stimulation at one level (i.e. cerebellar cortex, or Purkinje cells) or involve the entire cerebellum. This limited understanding is, however, not specific to cerebellar stimulation as similar questions remain regarding the effects of tDCS on the cerebral cortex.

### Aftereffects

Because transmembrane polarization, for even a few minutes, induces prolonged spiking activity in Golgi inhibitory cerebellar neurons (Hull and others 2013), Golgi cell changes may be a factor within the induced aftereffects of ctDCS. The mechanisms could involve ionic gradients in the extracellular space, inactivation or activation of specific cellular processes (including protein synthesis, gene expression, and channel or pump inactivation) common to various cell types (i.e., glia, smooth muscle cells in cerebellar vessels). ctDCS-induced changes in neurons could also involve receptors and neurotransmitters modulation. For instance, the main neurotransmitters in the cerebellum, γ-aminobutyric acid (GABA) and glutamate (Ottersen 1993), are both changed following tDCS applied over the sensorimotor cortex (Stagg and others 2009). Additionally, tDCS applied over slice preparations of mice M1 cortex paired with electrical stimuli results in LTP changes mediated by brain-derived neurotrophic factor (BDNF) and Tyrosine kinase B (TrkB) activation (Fritsch and others 2010). Whether similar or opposite effects (i.e., LTD) are present in the cerebellum is currently unknown. The cerebellum also contains myoinositol, a molecule also known to change in concentration after cerebral tDCS (Rango and others 2008). Thus, ctDCS-induced changes in cerebellar GABA, glutamate, BDNF, myoinositol, and other neurotransmitters may help explain its after effects (and possibly online effects). This suggestion is supported by the relatively slow time course in brain neurotransmitters changes after tDCS.

Finally, it is possible that cerebellar stimulation might act through non-neuronal mechanisms. For instance, electrical cerebellar stimulation increases cerebellar blood flow, which may affect synaptic activity in the cerebellar cortex (Iadecola and others 1997).

### Comparison with rTMS over Cerebellum

As noted above, single pulse TMS over cerebellum is often used to elicit activity in cerebellar output pathways, most notably that involved in CBI of motor cortex. In contrast, repeated pulses of TMS (rTMS) can produce after effects that are in many cases very similar to those observed after ctDCS. For example, an inhibitory rTMS protocol known as continuous theta burst stimulation (cTBS) can suppress CBI (Carrillo and others 2013) in the same way as cathodal ctDCS (Galea and others 2009); similarly, an excitatory rTMS protocol called intermittent theta burst stimulation (iTBS) can suppress PAS25 (Popa and others 2013) like anodal ctDCS (Hamada and others 2012). However, discrepancies can occur: rTMS protocols have sometimes been reported to alter the excitability of M1 to single pulse TMS (e.g., Oliveri and others 2005), whereas this has not been described after ctDCS (Galea and others 2009). The most likely explanation is that certain populations of cerebellar neurons share electrical characteristics such as orientation and size that make them equally likely to be targets of ctDCS and rTMS. Thus, the effects of each type of stimulation may well be similar. Presumably there are exceptions to this rule (non-neural structures may be more affected by DC fields than short duration TMS pulses) which mean that on occasion quite different effects may be observed.

At the moment, there are probably more studies of rTMS over cerebellum than ctDCS, particularly for clinical investigation and therapy. However, this may change in the future given that ctDCS is cheaper and simpler to apply. In addition, it has been shown in reduced preparations that DC electrical fields can promote axon growth. This may be another reason to promote its use in humans, although at the present time the ctDCS parameters for human work, such as intensity and duration of stimulation, are far lower than those used in basic science investigations.

## Investigations of ctDCS on Motor Function

### Adaptation of Reaching Movements

The ability of the motor system to adapt to changes in the environment is fundamentally important for the performance of accurate movements (Tseng and others 2007). Adaptive motor learning (adaptation) refers to situations where, in order to return to a former level of performance, an error stemming from an altered environment is reduced (Krakauer 2009). Neuropsychological studies have suggested that adaptation is a cerebellar-dependent process. For example, patients with cerebellar lesions show impaired adaptation (Martin and others 1996; Rabe and others 2009). In contrast, following adaptation the M1 is important for the retention of the new motor memory (Hadipour-Niktarash and others 2007; Tanaka and others 2009). Although this suggests a distinct role for the cerebellum and M1 during adaptation, this dissociation, until recently, had never been directly tested.

**Figure 4.**
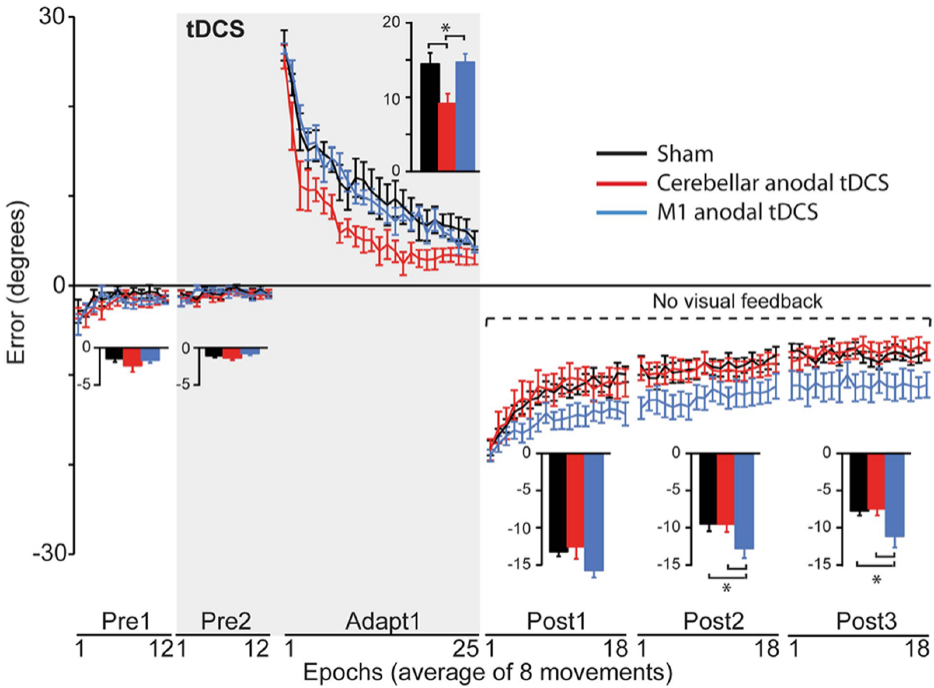
Results from Galea and others (2011). End-point error (degrees) are shown during baseline (Pre1, 2), adaptation (Adapt), and de-adaptation with no visual feedback (Post1, 2, 3) for the sham (black), CB (cerebellar anodal tDCS, red) and M1 (M1 anodal, blue) groups (mean ± SEM of 8 trial epochs). The shaded area represents blocks in which transcranial direct current stimulation (tDCS) was applied (Pre2, Adapt). Bar graphs insets indicate mean end-point error in degrees (±SEM) for Sham (black), CB (red), M1 (blue). For each block, separate one-way analyses of variance compared these values across groups. *P < 0.05. It is evident that anodal ctDCS led to faster acquisition (rapid error correction) whereas anodal M1 tDCS caused greater retention (longer presence of movements with the previously learned pattern).

A study by Galea and others (2011) sought to address the role of the cerebellum in adaptation using anodal tDCS. Participants made fast reaching movements while a 30° visuomotor transformation was introduced. During visuomotor adaptation, subjects received cerebellar, M1, or sham anodal tDCS. The authors found that ctDCS caused faster adaptation to the visuomotor transformation, as shown by a rapid reduction of movement errors (Fig. 4). This effect was not present with similar modulation of visual cortex excitability, suggesting that cerebellar modulation was the driving factor behind faster adaptation. In contrast, tDCS over M1 did not affect adaptation, but caused a marked increase in retention (Fig. 4). This demonstrates that the cerebellum and M1 have distinct functional roles and highlights the potential of tDCS as an effective tool to enhance cerebellar function (Galea and others 2011). Importantly, three recent studies have replicated these findings and shown that healthy older adults also experience beneficial improvements in adaptive motor learning when anodal ctTDCS is applied (Hardwick and Celnik 2014), that although visuomotor adaptation can be improved it does not alter intermanual transfer (Block and Celnik 2013), and that ctDCS also has a polarity specific effect when adapting to force field perturbations (Herzfeld and others 2014). These findings are clinically promising, as ctDCS might provide a useful approach to enhance stroke rehabilitation strategies that focus on adaptive learning such as robot-assisted therapy.

How can ctDCS enhance adaptation of reaching movements? It has been proposed that the cerebellum is crucially involved in adaptation learning because it forms a forward internal representation of movements that predicts the sensory consequences of motor commands (Miall and others 1993; Shadmehr and Krakauer 2008). The output of the commands forward model is sent to the parietal cortex where a prediction error is generated by comparing the predicted and actual sensory feedback. This prediction error is fed back to the cerebellum in order to modify the forward model. Within this framework, it is thought that the inhibitory output of the Purkinje cells is modulated by climbing fiber inputs transmitting the prediction error (Miall and others 1993; Shadmehr and Krakauer 2008; Tanaka and others 2009). As ctDCS is proposed to modulate Purkinje cell output (Galea and others 2009), it is possible that tDCS altered these cells response to the climbing fiber input. In other words, ctDCS improved adaptation by increasing Purkinje cell sensitivity to error.

### Effects of ctDCS on Walking Adaptation

Walking is a fundamental motor behavior that is controlled by interactions between spinal circuits, brainstem structures, the cerebellum, motor cortical areas and the basal ganglia. While the specific function of each of these areas is not fully understood, it is known that the cerebellum is of particular importance for adaptive learning of new walking patterns (Jayaram and others 2011; Morton and Bastian 2006). Similar to the reaching case previously described, adaptation is also defined here as the error-based learning process that occurs when we encounter predictable perturbations. It is a necessary process to maintain flexibility of the walking pattern in the face of new environmental demands.

Recent advances in brain stimulation have made it possible to assess cerebellar contributions to walking adaptation at a neurophysiological level in humans. CBI, a measure of connectivity between the cerebellum and M1, changes proportionally to walking adaptation (Jayaram and others 2011). Specifically, subjects were studied as they adapted their walking pattern on a split-belt treadmill where one leg had to walk three times faster than the other leg. Subjects initially take asymmetric steps, but adapt over hundreds of steps until their stepping is symmetric. CBI was assessed before and after subjects adapted the walking pattern on the split-belt treadmill. The change in CBI correlated with the amount of learning that occurred during split-belt walking. This study suggested that modulation in cerebellar excitability might affect adaptation of the walking pattern.

In a follow-up study, Jayaram and others (2012) investigated whether changing cerebellar excitability could change walking adaptation. Specifically, the authors tested whether there were polarity-specific effects of ctDCS on the rate or extent of locomotor adaptation. Anodal, cathodal, or sham ctDCS was applied to subjects as they adapted their walking pattern on a split-belt treadmill. Anodal ctDCS caused adaptation to occur more rapidly, whereas cathodal ctDCS slowed it down relative to sham stimulation. Neither anodal nor cathodal stimulation changed the adaptation amount or the de-adaptation rate or amount during washout. Interestingly, ctDCS affected the adaptation rate of learning spatial elements of walking (i.e., where to put the foot), but not temporal elements of walking (i.e., phase lags between leg motions). This suggests that stimulation of other brain or spinal sites might be necessary to affect the temporal elements of walking. The results of ctDCS are promising, as they might provide a useful tool to modulate therapies for walking training in people with gait impairments.

## Investigations of ctDCS on Cognitive Function

### Effects of ctDCS on Attention: Working Memory

Many activities require working memory (WM), a cognitive function for temporarily storing and manipulating visual/verbal information. Attention selectively controls information flow between WM modules, which affects cognitive resource allocation (Baddeley 1986). Recent studies support a role for the cerebellum in cognition, as evidenced by fMRI with WM-related activity in the lateral cerebellum and by impaired cognition in patients with cerebellar insults (Middleton and Strick 2000; Schmahmann 2004). Although care should be taken to exclude motor confounds when attributing such functions to the cerebellum, ctDCS can induce changes in cognition in healthy participants (Ferrucci and others 2008; Pope and Miall 2012). These early studies have lead researchers to believe that non-invasive stimulation could facilitate recovery in patients with impaired cognitive functions (Block and Celnik 2012; Pope and Miall 2014).

While tDCS of prefrontal cortex in healthy participants and stroke patients can enhance cognition (Fregni and others 2005; Jo and others 2009), stimulation of the cerebellum has received less attention so far, yet changes in cognitive performance after ctDCS have been shown. Ferrucci and others (2008) introduced the first study applying ctDCS while healthy individuals performed a modified version of the Sternberg item recognition task (i.e., identifying the presence/absence of a digit from a list of previously presented visual items, after a memory maintenance period). Fifteen minutes of stimulation over the entire cerebellum (irrespective of polarity) impaired the usual practice-dependent proficiency increase in this task (Ferrucci and others 2008). This finding was recently reproduced by Boehringer and others (2013). Although neither study found baseline performance to be enhanced by tDCS, Boehringer and colleagues showed that cognitive performance after ctDCS was affected by task difficulty/cognitive load, suggesting that the effects of ctDCS can be affected by the behavioral context.

**Figure 5.**
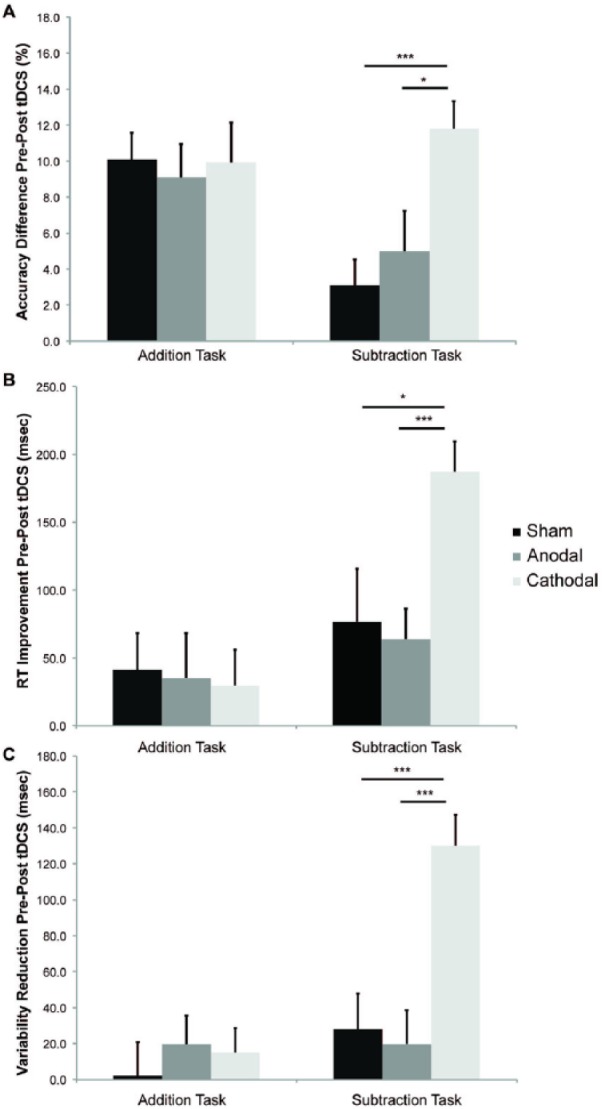
Changes in two working memory tasks (addition = PASAT and subtraction = PASST) expressed as percentage from session one (pre-stimulation) to session two (post-stimulation; n = 20). (A) This figure shows the gain in accuracy that participants’ experienced between stimulation sessions on the subtraction task, but not the addition task after cathodal stimulation only. (B) Improvement in response speed from pre-to post-stimulation. (C) Reduction in response latency variability. Participants performed calculations more quickly and paced them more consistently after cathodal, than after anodal or sham stimulation on the subtraction task only. Asterisks indicate significant differences (*P* < 0.05) as revealed with post hoc *t* tests (values are mean + 1 SEM). Modified from Pope and Miall (2012).

Task demand was also a feature of a recent study by Pope and Miall (2012) where cathodal tDCS over the right cerebellar hemisphere selectively enhanced performance during a demanding subtraction version of a mental arithmetic task (paced auditory serial subtraction test or PASST), but not during a simpler addition version (paced auditory serial addition test or PASAT; Figure 5). Since both tasks have similar motor demands, but a different cognitive load, Pope and Miall speculated that cathodal depression of the right cerebellar cortex might release cognitive resources by disinhibition of left prefrontal areas, as indicated by improvement in performance when cognitive demands are high. This finding and interpretation is supported by anatomical connections (Middleton and Strick 2000), as well as changes in functional connectivity between the cerebellum and frontal lobe regions in association to demanding tasks during mathematical cognitive exercise (Feng and others 2008). Indeed, as predicted by Pope and Miall’s work, anodal excitation of the left dorsolateral prefrontal cortex (DLPFC) selectively improves performance during the more demanding task, but not in the simpler math exercise (Pope and Miall, unpublished observation). These studies indicate that the understanding of cognitive enhancements in healthy participants must be constrained by cognitive and anatomical hypotheses regarding WM capacity and corticocerebellar connectivity.

fMRI results show that an increase in cognitive load is associated with enhanced neural activity in neocortical and lateral cerebellar areas (Salmi and others 2010). With this in mind, the excitatory effects of ctDCS could prove helpful in reducing cognitive decline in patients with marked dysfunction of the corticocerebellar pathway. Thus, future studies will need to address whether enhancing corticocerebellar connectivity by potentiating the reduced facilitatory drive from cerebellum to cerebrum can become an effective intervention for patients with cerebellar conditions.

### Effects of ctDCS on Sequence Learning

Recent studies investigated whether ctDCS affects sequence learning in a group of healthy volunteers performing the serial reaction time task (SRTT) (Ferrucci and others 2013). In this task, participants are unaware of the presence of a sequence, which is embedded within randomly presented stimuli. Participants also completed a visual attention task and a 100-mm visual analogue scale (VAS) for mood and fatigue. In the 21 subjects tested, anodal ctDCS improved sequence learning as measured by the SRTT. Importantly, the changes induced by ctDCS in SRTT performance were independent of arousal or alertness. This finding supports the concept that anodal ctDCS can improve cognitive function.

### Cerebellar tDCS and Language

The exploration of cerebellar contributions to language processing with tDCS remains in its infancy. To date, the nature of cerebellar contributions to language are elusive (De Smet and others 2013) and tDCS studies on language are hardly a decade old (see Monti and others 2013 for a review). Unlike cerebellar TMS (see Grimaldi and others 2014a for a review), there is only one published report of ctDCS effects on language (Pope and Miall 2012). In a verb-generation task, the rate and consistency of responses increased after cathodal right ctDCS, as compared to anodal ctDCS and sham tDCS. This effect was consistent with the mathematical findings described previously. Since this facilitation was also observed when anodal tDCS was applied over the cerebral cortex, the behavioural effects were interpreted as a consequence of disinhibition of the left DLPFC resulting from the inhibitory effect cathodal tDCS has on the overall inhibitory tone the cerebellum exerts over the cerebral cortex.

Predictions about ctDCS effects on language rely on the reciprocal connectivity of distinct cerebellar lobules with separate “motor” (M1, supplementary motor area [SMA]) and “non-motor” (DLPFC, pre-SMA) cerebral areas (Strick and others 2009), the findings that ctDCS modulates cerebellar–M1 connectivity (Galea and others 2009), the modulation of language functions by cerebral cortical tDCS (Iyer and others 2005) and the observation that ctDCS modulates aspects closely interfacing with language processing, for example, verbal WM (Ferrucci and others 2008) and procedural learning (Ferrucci and others 2013). Hence, since anodal tDCS over the left DLPFC improves verbal fluency (Iyer and others 2005) and reduces picture-naming latencies (Ferrucci and others 2010), facilitation is predicted after cathodal tDCS over right cerebellar loci. Similarly, since cathodal tDCS over the left DLPFC during recognition impairs verbal learning of auditorily presented nouns (Elmer and others 2009) and tDCS on the same area modulates performance in word memorization in later recognition (Javadi and Walsh 2012), anodal tDCS over right cerebellar loci would arguably induce similar impairments. Cerebellar tDCS may also yield effects on mechanisms of association, prediction and automatization in language processing, as already evidenced in cerebellar TMS studies on language (see Grimaldi and others 2014a for a review) and ctDCS studies on verbal WM (Ferrucci and others 2008). These predictions need to be tested in future studies.

## Cerebellar tDCS and Emotions

**Figure 6.**
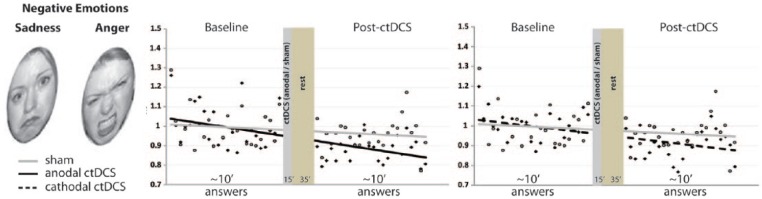
The figure shows the effect of cerebellar transcranial direct current stimulation (ctDCS) on negative emotional recognition (sadness and anger). *Y*-axis (arbitrary units, AU) represents trial to trial grand average of reaction times (RTs) across task stimulus presentation before and after ctDCS. *X*-axis represents answers, note that the *X*-axis graphically represents the time elapsing between the end of the task execution before stimulation and the beginning of the task execution after ctDCS. Note that anodal (left panel, solid line) and cathodal (right panel, dashed line) ctDCS both reduce baseline RTs for negative emotions. The trial-to-trial representation highlights the finding that anodal and cathodal curves differ from sham curves for negative emotions. Modified from Ferrucci and others (2013).

Among its non-motor roles, the cerebellum has been identified to be part of the network involved on processing complex emotional facial expression (Fusar-Poli and others 2009). In a recent study designed to understand the cerebellar role in emotional expression recognition, 21 healthy subjects were assessed before and after ctDCS (Ferrucci and others 2012). To investigate whether the ctDCS-induced effects depended on emotion recognition or reflected changes in arousal or attention, subjects rated their attention and mood in VAS. Anodal and cathodal ctDCS significantly enhanced the response to negative facial emotions leaving perception for positive and neutral facial expression unchanged (Fig 6). These findings suggest that the cerebellum plays a role in recognizing negative emotions, providing new insights into mental illnesses. Importantly, these early studies showing that ctDCS can affect responses in cognitive, emotional and learning tasks indicate that future investigations should address its potential as a tool to modulate complex mental processes.

## Can ctDCS Help Patients with Cerebellar Ataxias?

Cerebellar ataxias represent a heterogeneous and disabling group of sporadic and genetic diseases (Manto 2010), manifesting primarily with an impaired control of motion. However, it is now established that cerebellar symptoms extend beyond the pure motor deficits and include a wide spectrum of deficits in cognitive operations. We currently lack efficient drugs for the managements of most cerebellar ataxias. Neuromodulation of the cerebellum using ctDCS is therefore an exciting new development that has potential as a therapeutic strategy to reduce the symptoms resulting from cerebellar disorders.

A recent study conducted on a group of ataxic patients has assessed the effect of anodal ctDCS on (a) stretch reflexes in upper limbs, (b) upper limb dexterity and coordination using a mechanical counter test (MCT), and (c) computerized posturography (Grimaldi and Manto 2013). This investigation revealed that ctDCS significantly reduced the amplitude of long-latency stretch reflexes (LLSR), but did not improve the MCT scores or modify postural deficits. One possible explanation for these findings is that anodal ctDCS reduces the amplitude of LLSR by increasing the inhibitory effect exerted by the cerebellar cortex on cerebellar nuclei. The absence of effect on upper limb coordination and posture may suggest that the cerebellum–cerebral networks subserving these functions are less responsive to anodal ctDCS. Alternatively, it is possible that stimulating the surviving cerebellar neurons does not lead to sufficient changes in their activation since they might be already working at maximum capacity or the induced changes are not strong enough to compensate for the deficits.

A recent study in two patients with dominant spinocerebellar ataxia type 2 revealed that combining ctDCS and contralateral tDCS over M1 reduced both upper limb postural tremor and action tremor as well as dysmetria, an effect that was absent when sham stimulation was applied (Grimaldi and others 2014b). Interestingly, this study provided the first evidence that ctDCS followed by M1 tDCS exerts a favorable effect on the onset latency of the antagonist EMG activity associated with fast goal-directed movements toward aimed targets, a neurophysiological marker of the defect in programming of timing of motor commands (Grimaldi and others 2014b).

Taken together, these studies suggest that DCS has a potential therapeutic role in the symptomatic management of motor deficits in patients with cerebellar ataxias. However, important questions remain open. In particular, it is unclear whether there are subsets of cerebellar patients that are more or less responsive, how long the effects last, what is the optimal dose and whether deficits in cognitive operations could also be modified to a similar extent as compared with motor impairments. Finally, the extent of cerebellum volume loss in patients with cerebellar atrophy will likely influence the effectiveness of ctDCS, an issue that remains poorly understood.

## Safety Considerations about Cerebellar tDCS

To date no significant adverse events have been reported after the application of ctDCS. However, given the position and structure of the cerebellum particular care should be taken when targeting the posterior fossa with ctDCS. It is important to be aware that the brainstem might be affected by ctDCS, therefore studies applying it should consider monitoring vital signs as well as pain and discomfort. Additionally, whereas the suboccipital muscles of the neck present an issue in TMS studies of the cerebellum, ctDCS has the advantage of not inducing muscle twitches.

While tDCS is often described as a “safe” technique, it is important to note its safety limits. The primary limit to DC stimulation is the current density induced at the brain surface: A current density of 142 A/m^2^ is likely to damage neural tissue (Liebetanz and others 2009). With typical large-pad montages this current density is unlikely to be reached without incurring noticeable damage to the scalp. However, newer techniques such as high-density tDCS are able to deliver a more concentrated dose to the brain (Datta and others 2009).

Other important factors in tDCS safety are the interval between applications and the intensity. Interstimulation intervals of around 24 hours have led to long-lasting changes in behavior when applied to neocortical areas (Boggio and others 2007; Reis and others 2009). While this approach is appealing for the treatment of cerebellum-mediated disorders, it may provide an a priori indication of the minimum period that should separate sessions. Experimental applications of ctDCS have typically used higher levels of current (up to 2 mA) relative to cerebral cortical stimulation. This intensity is close to the maximum level of tolerability for many people, since tDCS at 2 mA frequently causes cutaneous sensations such as prickling, itching or mild pain or discomfort.

## Future Directions of ctDCS

Non-invasive brain stimulation techniques have achieved an important role in neuroscience because of their utility in the study of neurophysiological processes in alert behaving humans. In addition, clinical neurology and psychiatry are starting to adopt these techniques because of their potential application as therapeutic interventions (e.g., recent approval of repetitive TMS for migraine and depression management). ctDCS is an interesting approach that might promote functional and therapeutic changes in conditions where cerebellar activity is important. While cerebral tDCS has been tested extensively in the past 14 years, there is still limited research on the impact of cerebellar tDCS. One of the reasons for the relatively slow progress of ctDCS research is the limited neurophysiological data to guide clinical experiments. Indeed, the improvement in the understanding of the effects of tDCS on cortical areas has been facilitated by extensive neurophysiology research with TMS and or fMRI, which have assisted the search for optimal stimulation parameters. To date, the full extent of the benefits offered by ctDCS, both in research and rehabilitation, are unclear. Several issues need to be addressed before we will be able to consider ctDCS as a valuable therapeutic tool for neurological disorders.

Future investigations will need to address the exact mechanisms and area of influence of cerebellar tDCS to refine its application. To date, three studies using computer modeling current distribution showed that ctDCS main effects are focused on the cerebellum and its cortical structures (Parazzini and others 2013, 2014; Rampersad and others 2014). However, these studies need to be validated in animal models. This new knowledge will be useful to understand and modulate the interactions between cerebellar cortex and the deep nuclei, assuming that we can isolate and modulate their activity independently, for instance, during motor learning processes.

Cerebellar tDCS might also become a useful strategy to help patients with lesions in cerebral areas that are connected with the cerebellum. In these cases, rather than attempting to facilitate a partially damaged cortical region one could enhance cerebellar activity with the intention of facilitating the entire network. The difference in this approach would be that the port of entry to that network would be the intact cerebellum. As an illustration, patients with ideational and conceptual apraxia due to lesions in the temporal–parietal–occipital junction (Ochipa and others 1992), or ideomotor apraxia patients with left posterior parietal and or premotor cortex lesions (Manuel and others 2013; Rushworth and others 1997) might benefit from cerebellar stimulation because these abnormalities have a cerebellar component (Grealy and Lee 2011).

## Conclusions

The field of non-invasive brain stimulation, neurology and neurorehabilitation is experiencing an interesting new phase with the emergence of ctDCS. The currently available work shows that ctDCS can influence motor, cognitive and emotional behavior. This creates an exciting opportunity to develop this approach as a therapeutic intervention, especially for cerebellar ataxias, a group of disorders lacking effective alternative treatments. However, we are still in the process of understanding much of the properties of ctDCS and how best to apply this technique to human subjects. Future research will need to address the exact mechanisms underlying cerebellar tDCS, the optimal stimulation parameters, how it affects the physiology of the cerebellum and its safety.
